# HCV genotype-3a T cell immunity: specificity, function and impact of therapy

**DOI:** 10.1136/gutjnl-2011-300650

**Published:** 2012-11

**Authors:** Isla S Humphreys, Annette von Delft, Anthony Brown, Linda Hibbert, Jane D Collier, Graham R Foster, Monira Rahman, Annabel Christian, Paul Klenerman, Eleanor Barnes

**Affiliations:** 1 The Peter Medawar Building for Pathogen Research, University of Oxford, Oxford, UK; 2 Queen Marys, University of London, Institute of Cell and Molecular Sciences, The Liver Unit, London, UK; 3 John Radcliffe Hospital, Oxford, UK; 4 NIHR Oxford Biomedical Research Centre, Oxford, UK

**Keywords:** Hepatitis C virus, genotype-3a, T cell, adaptive immunity, interferon, ribavirin

## Abstract

**Background:**

Hepatitis C virus (HCV) genotype-3a infection is now the dominant strain in South Asia and the UK. Characteristic features include a favourable response to therapy; the reasons for this are unknown but may include distinct genotype-3a-specific T cell immunity. In contrast to genotype-1 infection, T cell immunity to this subtype is poorly defined.

**Objectives:**

The aims of the study were to (1) define the frequency, specificity and cross-reactivity of T cell immunity across the whole viral genome in genotype-3a infection and (2) assess the impact of interferon (IFN)-α/ribavirin on T cell immunity.

**Design:**

T cell responses in chronic and resolved HCV genotype-3a were analysed in comparison with genotype-1 infection (total n=85) using specific peptide panels in IFN-γ ELISpot assays. T cell responses were followed longitudinally in a subset of genotype-3a infected patients receiving therapy. Responses were further defined by CD4 and CD8 subset analysis, sequencing of autologous virus and cross-reactivity of genotype-3a with genotype-1a/-1b antigens.

**Results:**

CD8 T cell responses commonly targeted the non-structural (NS) proteins in chronic genotype-3a infection whereas in genotype-1 infection CD4 responses targeting HCV core predominated (p=0.0183). Resolved infection was associated with CD4 T cells targeting NS proteins. Paradoxically, a sustained response to therapy was associated with a brisk decline in virus-specific and total lymphocyte counts that recovered after treatment.

**Conclusion:**

HCV genotype-3a exhibits a distinct T cell specificity with implications for vaccine design. However, our data do not support the theory that genotype-3a viral clearance with therapy is associated with an enhanced antiviral T cell response. Paradoxically, a reduction in these responses may serve as a biomarker of IFN responsiveness.

**Significance of the study**

**What is already known about this subject?**

- Hepatitis C virus (HCV) genotype-3a is now the most prevalent HCV subtype in the UK and South Asia.
- Prophylactic and therapeutic vaccines are currently in development against genotype-1 infection. However, the T cell targets in genotype-3 infection are currently unknown.
- Interferon therapy has immunomodulatory properties; HCV genotype-3a is more responsive to interferon based therapies than HCV genotype-1. The effects of interferon on genotype-3a T cell immunity are not known.
- IL28B linked polymorphisms are associated with sustained virological response rates in HCV genotype-1 but not genotype-3a infections; therefore, alternative mechanisms that explain this observation should be explored.

**What are the new findings?**

- In contrast to genotype-1 infection, genotype-3a-specific CD8 T cell responses commonly target the non-structural hepatitis C virus (HCV) proteins in chronic disease.
- In chronic genotype-3a infection, T helper responses target a dominant HCV core protein.
- T cell targets identified during chronic infection may have a limited role in protective immunity since these differ from those found in spontaneously resolved infection where CD4 T cells targeting non-structural proteins dominate.
- Paradoxically, genotype-3a-specific T cell responses and total lymphocyte counts decline during interferon treatment in association with a sustained virological response.

**How might it impact on clinical practice in the foreseeable future?**

- Knowledge of genotype-3a-specific T immunity will aid rational vaccine design against this common subtype.
- The decline in T cells on treatment in association with viral decline may serve as an early biomarker of interferon responsiveness.

## Introduction

Hepatitis C virus (HCV) is a globally distributed pathogen that infects 3% of the world's population.^1^ Persistent infection may be associated with liver cirrhosis, hepatocellular cancer and death.^2^ HCV exhibits a high degree of genetic diversity and may be classified by phylogenetic analysis into seven major genotypes that share sequence homology of approximately 80% at the amino acid (aa) level and numerous subtypes.^3^ Phylogenetic analysis has shown that HCV has existed in human hosts for thousands of years, resulting in particular genotypes that are endemic in distinct geographical locations.^4^ However, over the last 100 years a number of distinct strains, in particular subtypes 1a, 1b and 3a, have become globally distributed in an epidemic that is associated with medical practice and intravenous drug use.

Since the introduction of screening for HCV in blood products in the UK, new genotype-1 infections have become less prevalent while genotype-3a infection has become relatively more common, especially among immigrant communities from the Indian subcontinent and the intravenous drug using population.^5^ Within the UK, the predominant strain is now genotype-3a.^6^ This subtype is also endemic in parts of Asia, Western Europe and is common (5%–10%) in the USA, although here genotype-1 remains the dominant strain.

The classification of HCV by viral genotype has proven to be highly informative in terms of the assessment of global viral evolution and epidemiology and in predicting the response to interferon (IFN) based treatment regimens. Large randomised clinical studies have consistently shown that genotype-3a has a more favourable treatment outcome than genotype-1 infection.^7^ The reason for this is not known, but may relate directly to genotype-specific viral sequence with a differential capacity to subvert the direct antiviral effects of IFN.^8^ ^9^ Recent data have demonstrated that host genetic polymorphisms linked to IFN-λ play a key role in determining sustained viral eradication using pegylated interferon (Peg IFN)/ribavirin therapy in genotype-1, but not in genotype-3 infection.^10^ ^11^ Alternatively, since IFNs are immunomodulatory,^12^ and genotypes-1 and -3 share limited sequence homology, the differential treatment outcomes may relate to the effects of therapy on genotype-specific T cell function or a distinct genotype-specific T cell repertoire in infected hosts, a hypothesis that is currently unexplored.

While the HCV-specific T cell response to genotype-1 infection has been extensively evaluated over the last decade, very little is known about the nature of the T cell responses that target other genotypes. While some studies have included patients with genotypes-2 and -3 infection, interpretation of these studies is confounded by the fact that immunological assays have relied almost entirely on genotype-1 peptides that do not represent the autologous circulating virus infection within the host. To date, analysis of genotype-3a-specific T cell responses is confined to a single study that assessed responses to the NS3 protein.^13^ This study, supported by our recent work assessing human leucocyte antigen (HLA) driven viral diversity between genotypes-1 and -3, has shown that there is likely to be limited T cell cross-reactivity between genotypes-1 and -3.^14^ ^15^

A detailed analysis of genotype-3a-specific T cell immunity across the entire genome has not hitherto been performed and is clearly important both in the context of rational vaccine development and in further understanding why the treatment outcome of IFN based regimens are genotype dependent. Since few HCV genotype-3a viral sequences are currently available within the major HCV databases, we first performed full-length sequence analysis in a genotype-3a infected cohort in order to define a robust and relevant consensus sequence, and designed a corresponding set of overlapping peptides for T cell analysis. Genotype-3a-specific T cell responses were then assessed in chronic and spontaneously resolved infection and during combination therapy, in association with autologous viral sequence.

## Methods

### Full-length genotype-3a genomic sequencing to design a genotype-3a peptide set and sequencing of autologous virus

Full-length (aa 1-2929) viral sequencing was performed on 20 treatment-naïve genotype-3a patients with chronic infection (John Radcliffe Hospital, Oxford) as previously described.^16^ In brief, plasma (500 μl) was concentrated by high-speed centrifugation (23 600 g for 1 h) at 4°C. Viral RNA was extracted using QIAmp Viral RNA Mini Kit (Qiagen, Crawley, UK). Reverse-transcription and first round PCR were performed in a single reaction (Superscript III Onestep RT-PCR system; Platinum Taq enzyme (Invitrogen, Grand Island, NY, USA)). First round reactions amplified a 4 kb product encoding Core, E1 and E2 structural proteins and a 7 kb product encoding the non-structural (NS2–5) proteins. Second round PCR used High Fidelity Taq DNA polymerase (Roche) in multiple nested PCR reactions (for sequences, see Humphreys *et al* 16 in addition to NS5B primers 8848-For 5′-TCC TGG TTR GGC AAC ATC ATC ATG TAC GC-3′ and 9428-Rev 5′-AAA TGG AGT GTT ATC CTA CCA GC-3′, and 9023-For 5′-GAC TCC ATG GTC TAA GCG CG-3′ and 9428-Rev). PCR fragments were gel purified (Qiagen) and sequenced bi-directionally using Prism Big Dye (Applied Biosystems, Warrington, UK) on an ABI 3100 DNA automated sequencer. Sequences were edited using X11 software. Accession numbers are GQ356200-GQ356215, GQ356217 and JF509175-JF509177.

A consensus sequence was determined to derive a genotype-3a peptide set (15–19 aa in length, overlapping by 11 aa, n=460) (Mimotopes, Australia). Subtype-1b J4 overlapping peptides and subtype-1a H77 (15–19aa) were obtained from BEI resources.^17^ All peptides with the C-terminal aa—G, P, E, D, Q, N, T, S, C—were either shortened or lengthened to ensure a tolerated aa at the end residue.^18^ Peptides were pooled into 10 pools corresponding to the individual viral proteins as follows: Core aa 1-191, E1 aa 192-383, E2 aa 384-752, p7 and NS2 aa 753-1032, NS3 protease domain aa 1033-1359, NS3 helicase domain aa 1349-1663, NS4 aa 1664-1978, NS5A aa 1979-2430, NS5B I aa 2431-2726 and NS5B II aa 2716-3021.

### Diversity of genotype-3a full-length sequence

Sequence diversity was assessed using full-length sequences from 20 chronic genotype-3a patients. A mathematical measure of entropy was determined for each amino acid position using the Shannon Heterogeneity In Alignments Tool V.1.0 (http://evolve.zoo.ox.ac.uk/software).^16^ A phylogenetic tree was constructed using MEGA5 programme according to the general time reversible method and bootstrap resampling set to 1000 replicates.^19^ Sequences were aligned using the ClustalX V.2.0.12 programme.

### Clinical cohort

Eighty-five treatment-naïve patients, 44 chronic genotype-1a/1b, 31 chronic genotype-3a and 10 spontaneously resolved infection (HCV Ab+ RNA negative), were recruited (John Radcliffe Hospital, Oxford, and Barts and the London NHS Trust, London, UK). A subset of 21 genotype-3a patients were assessed immediately pre-treatment, during and after therapy (peg IFN-α2b, 180 μg/week and ribavirin 800–1200 mg/day dependent on body weight given for 24 weeks). Local ethical approval was obtained and all patients gave written informed consent. Treatment response is defined using standard definitions.^7^

### ELISpot assays

Peripheral blood mononuclear cells (PBMC) were isolated and frozen immediately. Frozen PBMC enabled the concurrent assessment of T cell responses sampled at different time points. Thawed PBMC were tested by IFN-γ (Mabtech, Nacka Strand, Sweden) ELISpot assays, as previously described.^20^ Briefly, viable PBMC (200 000/well) plated in duplicate were stimulated for 18 h with peptide pools A-M (3 μg/ml), cytomegalovirus (CMV) lysate (0.05 μg/ml, Chiron), influenza, Epstein-Barr virus (EBV) and CMV (FEC) CD8 epitopes in a single pool (3 μg/ml BEI resources). Spot-forming units (SFU) were counted using an automated ELISpot plate reader (AID). For the genotype-3a ELISpot, a positive cut-off of 40 SFU/10^6^ PBMC was defined in 12 healthy volunteers using: (mean SFU/10^6^PBMC in test wells - negative control wells)+ 3×SD. For genotype-1, the cut-off of 43 SFU/10^6^ was previously defined in healthy volunteers using an identical strategy. Total lymphocyte counts were assessed from 200 μL of blood pre-treatment and at treatment week 12 using the Sysmex Automated Hematology Analyzer XE-2100 (Sysmex Corporation, Milton Keynes, UK).

### T cell subset analysis and fine mapping of antigenic targets

To define T cells subsets, CD8 T cells were depleted from PBMC using magnetic bead separation (CD8 Dynabeads, Invitrogen) following manufacturer's instructions. The CD8 negative PBMC were used in IFN-γ ELISpot assays to map antigenic targets.

### T cell lines and ICS

PBMC (5×10^6^) were stimulated with 2.5 μg/ml antigen supplemented with 50 IU/ml recombinant interleukin 2 (days 2, 5 and 8). After 10–14 days of culture, T cell lines were rested for 24 h. Intracellular cytokine stains (ICS) staining of PBMC ex vivo and in short-term stimulated cell lines was performed as previously described (Colloca *et al* 21). In brief, PBMC were stimulated using specific HCV peptides or PMA/Ionomycin. Unstimulated cells served as the negative control. After 6 h, cells were permeabilised and stained using the following antibodies: CD3-PO, CD4-Qdot 605, CD8-PB, IFNγ-Alexa-Fluor700, IL2-APC, TNFα-PE-Cy7 and Mip1beta-PE. Flow cytometry was performed using a BD LSRII and analysis was by FlowJo (V.8.8.6).

### Statistical analysis

T cells targeting structural and NS viral genomic regions in genotypes-3 and -1 HCV infected patients used the Fisher's exact test. The magnitude of the T cells response in chronic versus spontaneously resolved infection, and pre-treatment in relation to treatment outcome was assessed by the unpaired two-tailed t test. Comparisons between T cell responses over time used the paired t test. A p value <0.05 was considered significant.

## Results

### T cells in genotype-3a infection predominantly target NS proteins

**Figure 1:**
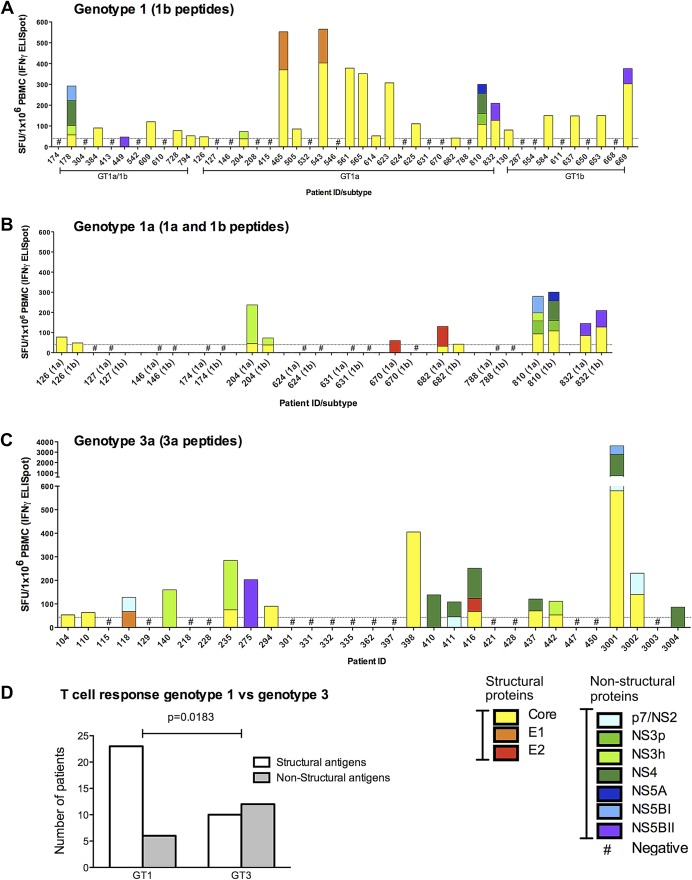
Hepatitis C virus (HCV)-specific interferon (IFN)-γ T cell responses in genotypes-1 and -3 chronic infection. The total magnitude of the HCV-specific T cell responses measured by IFN-γ ELISpot assay (spot-forming units (SFU)/10^6^ peripheral blood mononuclear cells (PBMC)) in (A) genotype-1 chronically infected patients using genotype-1b peptides, (B) a subset of chronically infected genotype-1a patients using genotypes-1a and 1b peptides and (C) genotype-3a patients using genotype-3a peptides. T cell responses to distinct parts of the viral genome are colour coded. (D) A comparative analysis of patients with genotype-3a or -1 chronic infection targeting structural and non-structural viral regions as assessed by IFN-γ ELISpot is shown (p=0.0183).

We have previously shown in chronic genotype-1 infection that HCV-specific CD8 T cells responses are rarely detected ex vivo, though CD4 T cells targeting multiple core epitopes are frequently observed.^22–27^ This study confirmed the previous observations; in genotype-1 infection ex vivo T cell responses targeting HCV core were frequently detected (23/44 patients), while weak responses targeting the NS proteins could be seen in only 6/44 patients (figure 1A). In a subset of 12 genotype-1a patients, T cell responses were assessed using both a genotype-1a and a genotype-1b peptide panel. Responses to the NS proteins were rarely detected irrespective of the peptide panel used (figure 1B). In contrast, T cells targeting the NS region were readily detectable and significantly more common in genotype-3 infection (6/44 genotype-1 vs 12/31 genotype-3 infected patients p=0.0183) (figure 1C,D). Additional responses targeting HCV core could be detected in 10/31 genotype-3a infected patients (figure 1C).

### Mapping of genotype-3a antigenic targets in chronic infection

**Figure 2:**
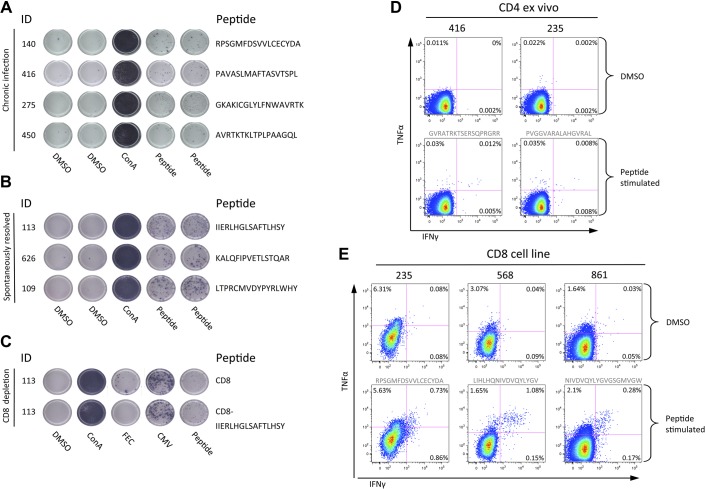
T cell responses assessed by interferon-γ ELISpot and intracellular cytokine stains (ICS). Representative T cell responses detected by interferon-γ ELISpot assay in (A) chronic hepatitis C virus (HCV) infection and (B) spontaneously resolved infection. An example of a CD8 T cell depletion ELISpot is shown (C). Ex vivo ICS analysis of CD4 responses to two core peptides in chronic patients (peptide sequence in grey text) (D). ICS following the generation of short term cells lines in chronically infected (pt 235) and spontaneous resolved infection (pt 568, and 861) (E). CMV, cytomegalovirus; TNF, tumour necrosis factor.

T cell antigenic targets were identified using single peptides in IFN-γ ELISpot assays (table 1). CD4:CD8 subclass was defined using CD8 IFN-γ ELISpot depletion assays, and confirmed with ex vivo ICS, or with ICS following the generation of short-term cell lines (figure 2). In 11/15 patients with detectable responses and cells available, 10 genotype-3a T cell epitopes were identified of which eight are novel, including a dominant CD4 response to HCV core (aa 143-158 PVGGVARALAHGVRAL) (in four patients with responses at the threshold of detection responses could not be mapped). T cell epitopes that mapped to the NS viral regions NS3 (aa 1513-1529 RPSGMFDSVVL), NS4b (aa 1791-1806 PAVASLMAFTASVTSPL, 1824-1841 THLAGPQSSSAFVVSGLA and 1918-1933 EGAVQWMNRLIAFASR), NS5a (2029-2046 GVMSTRCPCGASIAGHVK) and NS5b (2946-2963 GKAKICGLYLFNWAVRTK and 2965-2975 KLTPLPAAGQL) were exclusively CD8 T cell restricted. CD8 responses targeting a single epitope in NS3 helicase (aa 1513-1529 RPSGMFDSVVL) were found in all three HLA B3501 patients (the equivalent viral region in genotype-1 infection RPSGMFDS***S***VL is a known B3501 epitope).^13^

**Table 1:** T cell epitopes in genotype-3a infection

Protein	Amino acid	3a Peptide sequence used in T cell assays	Patient no	Patient viral sequence pre-treatment	HLA	Treatment outcome	Defining CD4:CD8 T cell subsets
Core	27–44	GGQIVGGVYVLPRRGPRL	362	GGQIVGGVYVLPRRGPRL	DRB1 0103, 0701	SVR	NA
	73–90	GRSWAQPGYPWPLYGNEG*	416	GRSWAQPGYPWPLYGNEG	DRBI 0101, 0701 DQBI 0201, 0501	SVR	NA
	143–158	PVGGVARALAHGVRAL*	110	P**A**GGVARALAHGVRAL	DRB1 0801, 1101	SVR	CD4
			331	PVGGVARALAHGVRAL	NA	SVR	
			437	PVGGVARALAHGVRAL	DRB1 0103, 0501	SVR	
NS3 helicase	1513–1529	RPSGMFDSVVL* †	140	RPSGMFDSVVLCECYDA	A2402, A3002, B0702, B3501	SVR	CD8
			235	RPSGMFDSVVLCECYDA	A0301, A3002, B0702, B3501, C0401, C0702	SVR	
			362	RPSGMFDSVVLCECYDA	A2402, B3501, B4402, C0401, C0409	SVR	
NS4b	1791–1806	PAVASLMAFTASVTSPL*	416	PAVASLMAFTASVTSPL	A0101, A0201, B0801, B5701, C0602, C0701 DRB1 0101, 0701	SVR	CD8
			437	NA	A0201, A2601, B3801, B2702, C1203, C0102 DRB1 0103, 0101	SVR	
	1824–1841	THLAGPQSSSAFVVSGLA*	437	NA	A0201, A2601, B3801, B2702, C1203, C0102 DRB1 0103, 0101	SVR	NA
	1918–1933	EGAVQWMNRLIAFASR‡	410	EGAVQWMNRLIAFASR	A0101, A3001, B1302, B4402, C0602, C0501 DRB1 0701, DQB1 0201	NR	CD8
NS5a	2029–2046	GVMSTRCPCGASIAGHVK*	129	GVMSTRCPCGASI**T**GHVK	A1101, A7401, B4403, B38, C04, C0702	REL	CD8
NS5b	2946–2963	GKAKICGLYLFNWAVRTK*	275	GKAKI**T**GLYLFNWAVRTK	A1101, B0702, B4402, C0501, C0702 DRB1 0401, 0407	SVR	CD8
	2965–2975	KLTPLPAAGQL* †	450	KLTPLPAAG**L**L	NA	REL	CD8

Peptide amino acid sequence and patient viral sequence from pre-treatment time point are shown (amino acid differences between these are shown in bold).

CD4:CD8 T cell subset analysis was assessed using PBMC in IFN-γ ELISpot assays following CD8 depletion.

* Epitopes where viral sequence is different between genotypes-3a and -1.

† Peptides restricted to 11 residues in ELISpot assays based on previously published epitope or the overlapping sequence between two adjacent immunogenic peptides.

‡ Epitopes where viral sequence is identical between genotypes-3a and -1a.

IFN, interferon; NA, not available (PCR amplification not possible or insufficient sample); PBMC, peripheral blood mononuclear cells; REL, relapse; SVR, sustained virological response; NR, non-responder.

Viral sequence analysis was used to determine whether the host circulating viral sequence represented the identified T cell antigenic targets. In 7/11 cases the host viral sequence was identical to the peptide T cells antigenic target. However, amino acid differences between host viral sequence and peptide targets were found in four patients (table 1). In two patients (129 and 450 targeting peptides NS5a aa 2029-2046 and NS5b aa 2965-2975, respectively), T cell analysis using variant and consensus peptides in IFN-γ ELISpot assays showed that the variant peptide resulted in a reduction or total loss of T cell response.

### Assessment of genotype-3a viral diversity

**Figure 3:**
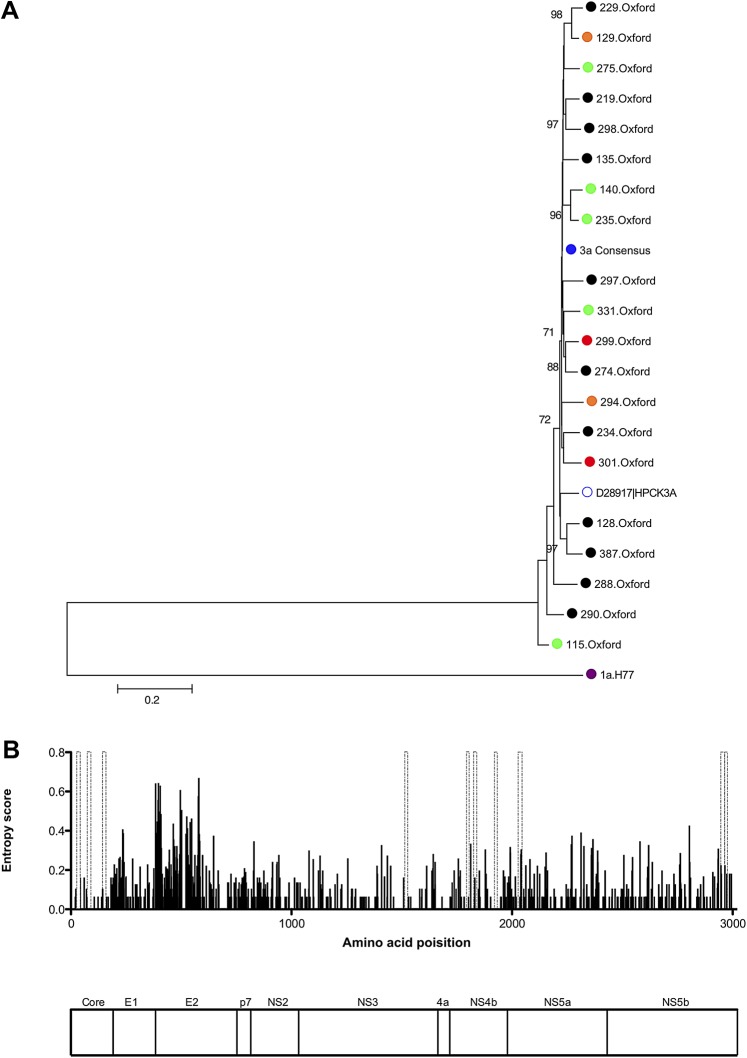
Sequence diversity of full-length genotype-3a sequences. (A) Neighbour-joining tree of full-length nucleotide sequences from 20 genotype-3a infected chronic patients, including eight patients followed longitudinally through combination therapy (green = sustained virological response, orange = relapse, red = non-responders, genotype-3a consensus sequence = blue). Also included are the genotype-3a peptide consensus sequence, a genotype-3a reference sequence (accession number D28917), together with H77 genotype-1a nucleotide sequence (accession number AF009606) used as an outgroup. Bootstrap scores >70% are shown. (B) Entropy score (measure of viral variability) across the viral genome using full-length genotype-3a sequences from 20 chronic genotype-3a patients is shown. Genotype-3a peptides positively identified by interferon-γ ELISpot assays in chronic disease are indicated by dashed grey bars. A map of hepatitis C virus polyprotein that corresponds to the entropy plot above is shown.

To ensure that the local Oxford cohort did not represent a single outbreak and so account for the readily detectable CD8 responses in the NS region using consensus peptide (figure 3A), and since there are limited data on genotype-3a viral sequence, viral diversity was assessed in the genotype-3a cohort using full-length viral sequences. Phylogenetic analysis showed significant diversity and a genotype-3a reference strain (accession number D28917) fell within the Oxford genotype-3a cluster. The entropy map showed that significant viral variation was observed throughout the viral genome, particularly within E2 but also in the NS regions (figure 3B).

### Genotype-3a-specific T cell responses in spontaneously resolved infection

**Figure 4:**
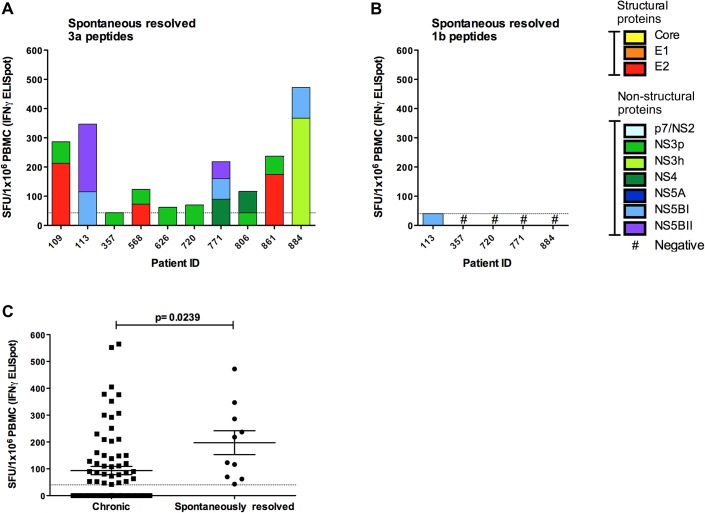
Hepatitis C virus (HCV)-specific interferon (IFN)-γ T cell responses in spontaneously resolved infection. The total magnitude of HCV-specific T cell responses measured by IFN-γ ELISpot assay (spot-forming units (SFU)/10^6^ peripheral blood mononuclear cells (PBMC)) in patients with spontaneously resolved HCV infection using (A) genotype-3a and (B) genotype-1b HCV peptides. T cell responses to distinct parts of the viral genome are colour coded. (C) A comparative analysis of the magnitude of responses of patients with chronic infection and spontaneous resolvers as assessed by IFN-γ ELISpot is shown (p=0.0239).

In spontaneously resolved infection, T cell responses (assessed by IFN-γ ELISpot/CD8 T cell depletion and ICS assays; figure 2) were detected in all individuals with resolved infection (10/10 resolved vs 16/32 chronic, p=0.0045) (figure 4A). The total mean magnitude was significantly higher compared with chronic infection (197.4±44.50 resolved, 139.8±48.70 chronic, p=0.02 (pt 3001 outlier excluded)) (figure 4C). In contrast to responses observed in chronic disease responses to NS proteins were CD4 T cell restricted, and the HCV CD4 core response that was dominant on chronic infection was not detected. No responses were detected in a subset of five patients who were also tested with a genotype-1b peptide set (figure 4B) demonstrating limited inter-genotypic cross-reactivity. T cell antigenic targets in patients with resolved infection are detailed in table 2.

**Table 2:** Genotype-3a peptides identified in spontaneous resolvers

Protein	Amino acid	3a Peptide sequence*	Patients	1b Peptide sequence	Outcome	Defining CD4:CD8 T cell subsets
E2	610–625	LTPRCMVDYPYRLWHY	109	LTPRC**L**VDYPYRLWHY	SR	NA
	696–712	LIHLHQNIVDVQYLYGV	568	LIHLHQNIVDVQYLYG**I**	SR	CD8†
	702–719	NIVDVQYLYGVGSGMVGW	861	NIVDVQYLYGVGS**AF**V**SF**	SR	CD8†
NS3 protease	1198–1213	KALQFIPVETLSTQAR	626	KA**VD**F**V**PVE**SME**T**TMR**	SR	CD4
	1288–1305	GNRTVTTGAKLTYSTYGK	109	G**VR**T**I**TTGA**PV**TYSTYGK	SR	NA
NS3 helicase	1423–1440	AYYRGLDVSVIPTAGDVV	884	AYYRGLDVSVIPT**I**GDVV	SR	CD4
NS4b	1805–1822	TSPLTTNQTMFFNILGGW	806	TSPLTT**QS**T**LL**FNILGGW	SR	NA
NS5b	2548–2565	NQIRSVWEDLLEDTTTPI	113	N**H**I**H**SVW**K**DLLEDT**V**TPI	SR	CD4
	2603–2618	KRALYDVIQKLSIETM	884	K**M**ALYDV**VST**L**PQVV**M	SR	CD4
	2893–2908	IIERLHGLSAFTLHSY	113	IIERLHGLSAF**S**LHSY	SR	CD4

* Genoype 3a peptide sequence identified as T cell targets in patients with resolved infection. Sequence differences from genotype-1b peptides is shown in bold.

† CD4:CD8 T cell subset analysis assessed using ICS assays.

CD4:CD8 T cell subset analysis was assessed using PBMC in IFN-γ ELISpot assays following CD8 depletion.

ICS, intracellular cytokine stains; IFN, interferon; PBMC, peripheral blood mononuclear cells.

### Cross-reactivity of genotype-3a and -1 T cell targets

Our previous work using a viral sequenced based approach in association with HLA type predicted little cross-reactivity between CD8 T cells that target genotypes-1 and -3.^28^ However, this methodology can only identify T cell targets that are subject to HLA-restricted viral escape. In contrast, consensus peptides are likely to detect relatively conserved antigenic targets. In all cases where cross-reactivity was assessed the equivalent viral region in genotype-1 infection differed from the genotype-3 T cell epitopes (table 3). Using peptides derived from the equivalent regions in genotype-1, we show that in all cases (with the exception of NS5a aa 2029-2046) there was a total loss or reduction in T cell recognition. In the KLTPLPAAGQL NS5b genotype-3a epitope where the host circulating viral variant (KLTPLPAAG**L**L, table 1) led to loss of recognition, the equivalent genotype-1 viral regions differed from the genotype-3a sequence at the same residue (KLTPIAAAG**R**L and KLTPLIAAG**S**L) and inter-genotypic cross-recognition was abolished.

**Table 3:** Genotype-3a and -1 peptide cross-reactivity

Protein	3a Amino acid	3a Peptide	3a Peptide SFU/10^6^ PBMC	1a And/or 1b equivalent sequence		1a/1b Peptide SFU/10^6^ PBMC	3a vs 1a/1b Cross-reactivity
Core	73–90	GRSWAQPGYPWPLYGNEG	33	1a	GR**T**WAQPGYPWPLYGNEG	28	↓
				1b	GR**A**WAQPGYPWPLYGNEG**L**	23	↓
	143–158	PVGGVARALAHGVRAL	50	1a = 1b	**GA**P**L**G**A**VARALAHGVR**V**L	23	↓
NS3 helicase	1423–1440	AYYRGLDVSVIPTAGDVV	267	1b	AYYRGLDVSVIPT**I**GDVV	0	↓
	1513–1523	RPSGMFDSVVL	30	1a =1b	RPSGMFDS**S**VL	8	↓
NS4b	1791–1806	PAVASLMAFTASVTSPL	65	1a	PA**I**ASLMAFTA**A**VTSPL	0	–
				1b	PA**I**ASLMAFTAS**I**TSPL	0	–
NS5a	2029–2046	GVMSTRCPCGASIAGHVK	23	1a	**I**V**H**STR**H**PCG**E**S**T**AGHVK	30	↑
				1b	G**I**M**Q**T**T**CPCGA**Q**IAGHVK	13	↓
NS5b	2603–2618	KRALYDVIQKLSIETM	100	1b	K**M**ALYDV**VST**L**PQVV**M	8	↓
	2965–2975	AVRTKTKLTPLPAAGQL	33	1a	VRTK**L**KLTP**IA**AAG**R**LDL	0	–
				1b	AVRTK**L**KLTP**I**PAA**S**QL	5	↑

T cell responses were assessed in subtype-3a patients using the positively identified subtype-3a peptides and the equivalent 1a and 1b peptides (IFN-λ ELISpot assays). Amino acids that differ from the subtype-3a peptides are shown in bold.

– loss of cross reactivity, ↓ decrease in reactivity, ↑ increase in reactivity.

IFN, interferon; PBMC, peripheral blood mononuclear cells; SFU, spot-forming units.

Although DRB1*0103 and DRB1*1101 restricted CD4 T cells targeting aa 143-158 have been previously described in genotype-1 infection,^29–31^ there is significant sequence divergence between genotypes-1 and -3 at this region (PVGGVARALAHGVRAL vs GAPLGAVARALAHGVRVL). Here the dominant CD4 T cells response targeting the genotype-3a core epitope (aa143-158) was found to be relatively genotype-3a-specific in that the equivalent genotype-1 viral region was poorly recognised.

### Genotype-3a HCV-specific T cell responses decrease during combination therapy

**Figure 5:**
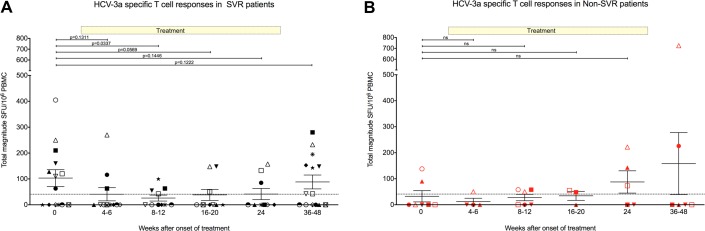
Effect of combination therapy on hepatitis C virus (HCV)-specific T cell responses in genotype-3a patients with sustained virological response (SVR) (A) and non-SVR (B). Total magnitude of genotype-3a HCV-specific T cell response measured by IFN-γ ELISpot assay in chronic genotype-3a patients. T cell responses to HCV genotype-3a peptides were measured before treatment, and at multiple timepoints during treatment and post-treatment. Patients achieving SVR are shown on left panel (black symbols) and patients failing to achieve an SVR are shown on right panel (red symbols). Each patient is represented by a different symbol. Threshold of positive HCV-specific responses is represented by dotted line (40 spot-forming units (SFU)/10^6^ peripheral blood mononuclear cells (PBMC)) defined in healthy controls (see Methods). Treatment duration of 24 weeks is shown.

Twenty-one patients infected with genotype-3a were followed longitudinally during standard treatment with 6 months of PEG-IFN-α and ribavirin to test the hypothesis that therapy may enhance genotype-3a-specific T cell immunity and account for the preferential treatment response rates in this genotype (figure 5). Of the 21 patients, 14 (67%) achieved sustained virological response (SVR), 5 (24%) experienced virological relapse and 2 (9%) were non-responders (NR). Pre-treatment, 8/14 SVR patients had detectable HCV-specific T cell responses with a mean total magnitude of 180 SFU/10^6^ PBMC (figure 5A). This was higher than that observed in the non-SVR patients, where responses were seen in only two patients with a mean total magnitude of 32 SFU/10^6^ PBMC T cell responses (p = NS, figure 5B).

In the SVR patients, the total HCV-specific T cell response detected pre-treatment clearly declined during therapy (mean pre-treatment 180 SFU/10^6^ PBMC vs 26 SFU/10^6^ PBMC at weeks 8–12; p=0.0337) (figure 5A). In general, responses increased again after treatment when virus remained undetectable suggesting that the decline was not simply due to decreasing levels of viraemia.

In three patients, new weak HCV-specific T cell responses were first detected 3–6 months after the end of therapy (2 SVR and 1 non-SVR patients). This phenomenon has been observed in genotype-1 infection with an increase in proliferative and cytotoxic capacity in both SVR and non-SVR patients towards the end of therapy and seems to be unrelated to treatment outcome.^30^ ^32^

### Other virus-specific T cell responses decline during IFN and ribavirin therapy

To determine if the decrease in magnitude of T cell responses during treatment applied to HCV-specific T cells only, T cell responses to other viral antigens (influenza, EBV and CMV HLA CD8 T cell restricted epitopes, and CMV lysate that primarily induce CD4 T cells) were assessed.

**Figure 6:**
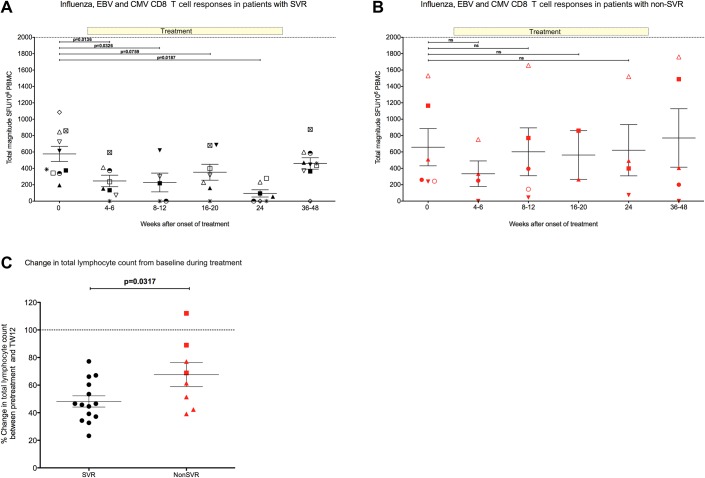
Effect of combination therapy on influenza, Epstein-Barr virus and cytomegalovirus (CMV)-specific (FEC) T cell responses and the total lymphocyte count in sustained virological response (SVR) and non-SVR patients. Total magnitude of FEC and HCV specific IFN-γ T cell responses measured by ELISpot assay in chronic genotype-3a patients. Responses to FEC HLA class-I restricted peptides were measured before treatment, at multiple timepoints during treatment and post-treatment. (A) Patients achieving SVR shown on left panel (black symbols) and (B) patients failing treatment are shown on right panel (red symbols). Threshold of positive hepatitis C virus (HCV)-specific responses is represented by dotted line (40 spot-forming units (SFU)/10^6^ peripheral blood mononuclear cells (PBMC)) defined in healthy controls (see Methods). Treatment duration of 24 weeks is shown. (C) Percentage change in pre-treatment lymphocyte count by week 12 of combination therapy in 22 patients chronically infected with HCV genotype-3a. Pre-treatment level set at 100% is represented by dotted line. Patients achieving an SVR by 6 months post-treatment are shown by black circles, relapse patients shown by red triangles and non-responders shown by red squares. Statistical significance measured by unpaired t test indicated by p value <0.05. Non-SVR=relapse and non-responder patients.

Pre-treatment influenza/EBV/CMV responses were detected in 16 patients (10 SVR, 5 REL, 1 NR-magnitude, range 195–1530 SFU/10^6^ PBMC) (figure 6). In SVR patients these decreased in magnitude throughout treatment and were significantly lower at all time points during treatment compared with pre-treatment (magnitude at TW4-6, TW8-12 and TW24 vs pre-treatment p=0.0136, p=0.0326, p=0.0187, respectively) (figure 6A). Following treatment, the magnitude of the responses increased to pre-treatment levels. In contrast, the responses of the NR patients did not change significantly during treatment (figure 6B). Similarly, the pre-treatment CMV lysate responses also decreased in magnitude during treatment in the SVR patients but did not reach statistical significance (data not shown).

### SVR with IFN-a and ribavirin is associated with total lymphopenia

As the magnitude of the T cell responses to HCV, influenza, EBV and CMV decreased during treatment in eight SVR patients, the effect of treatment on total lymphocyte number in 21 genotype-3a patients were assessed (14 SVR, 5 REL, 3 NR). The magnitude of the pre-treatment total lymphocyte count was not associated with treatment outcome (data not shown). The effect of treatment on lymphocyte count was assessed by measuring the percentage change in the pre-treatment total lymphocyte count by treatment week 12 (figure 6C). The total lymphocyte number decreased by 50% in SVR and relapse patients, but decreased to only 90% of pre-treatment levels in NR patients (SVR vs NR p=0.0010).

## Discussion

While T cell immunity to HCV genotype-1 infection has been extensively studied and appears to play a key role in clinical outcome, very little is known about T cell immunity to other viral subtypes. This study focuses on HCV genotype-3a infection, now the dominant infecting subtype in the UK and large parts of Asia,^33^ and assesses T cell immunity targeting the entire genotype-3a polyprotein in association with viral sequence analysis and treatment outcome. Since IFNs are known to modulate T cell function, and since viral homology between different genotype approximates to 80%, it is plausible that IFNs induce a distinct T cell response in genotype-3a infection, explaining at least in part the distinct treatment outcomes.

Since very little genotype-3a viral sequence data were available for major parts of the polyprotein, we initially performed full-length viral sequence analysis in 20 treatment-naïve genotype-3a infected patients. This enabled the design of a representative genotype-3a peptide set. We found that HCV-specific T cell responses were readily detectable in approximately half the patients with chronic genotype-3a infection, with CD4 T cell subsets targeting the core protein and CD8 T cells targeting the NS HCV proteins. This was significantly different to genotype-1 infection, where detectable responses almost exclusively targeted core. This may be explained in part by the mixed 1a/-1b-subtype populations, although testing with homologous 1a/1b peptide panels did not increase the detection of NS responses.

The genotype-3a CD4 core responses were mapped down to aa 143-158; PVGGVARALAHGVRAL in 4/5 cases. The ‘equivalent’ genotype-1a peptide, which differs by two amino acids, is known to be a DRB1 1101 restricted CD4 T cell target.^31^ In contrast, in genotype-1 infection, CD4 T cells are known to target a broad range of epitopes within core.^23^ ^24^ ^34^ Viral sequence analysis in 4/5 patients showed no evidence of viral escape supporting previous data in humans,^23^ and chimpanzees,^35^ showing that CD4 T cells unlike CD8 T cells rarely exert selection pressure on HCV. In resolved infection, the core responses were not detectable suggesting that these may represent low avidity responses that emerge once chronic infection is established.

Where T cell subset analysis was possible, we showed that genotype-3a NS proteins were targeted exclusively by CD8 T cells in chronic infection, whereas in resolved infection CD4 T cells were also detected. This observation is in line with previous studies that demonstrate the importance of the CD4 subset in viral control.^31^ Intrahepatic T cell immunity has not been assessed; while T cells assessed in the peripheral compartment are thought to reflect intrahepatic T cell immunity, higher levels of HCV-specific T cells are seen in the liver and we cannot rule out the possibility that T cell subsets within the liver differ between genotypes.^36^ ^37^

Nine genotype-3a epitopes targeting core and NS3-NS5b are described in chronic infection. Interestingly, four (NS4b 1791-1806, NS4b 1918-1933, NS5b 2946-2963, NS5b2965-2975) of these identified as CD8 T cell epitopes overlap with CD4 T cell epitopes in the non-identical but equivalent genotype-1a viral regions.^29^ ^31^ ^38^ ^39^ Only in one epitope was the genotype-3a viral sequence identical to that found in genotype-1a infection. The majority of the genotype-3a epitopes assessed for cross-reactivity showed little or no cross-reactivity with the equivalent genotype-1 antigens, showing that the majority of T cell targets in genotype-3a infection are subtype-specific.^13^

Next, we assessed the effect of combination therapy on genotype-3a-specific responses. The effect of therapy on HCV-specific T cell responses has been an area of investigation and controversy for some years in genotype-1 infection but is unexplored in genotype-3a infection with the exception of one study suggesting that proliferative responses to NS3 are enhanced in genotype-3 infection during treatment.^40^ How exactly therapy impacts on anti-viral T cell responses is complicated by the fact that IFN has both direct antiviral and also a broad range of immunomodulatory properties. Disparate results in genotype-1 infection may be accounted for by technical challenges such as weak T cell responses at the detection limit of immunological assays, and the fact that peptides have been used in assays that do not reflect the circulating viral strain. Studies have shown that T cell responses undetectable before therapy can be detected during therapy at least in some individuals,^30^ ^41^ that the generation of a Th1 type response during therapy is associated with an SVR^42^ and that the level of pre-treatment HCV-specific immunity is associated with an SVR.^32^ ^43^ In support of the latter, we noted that the total HCV-specific T cell responses pre-treatment were higher than in NR patients; however, this did not reach statistical significance. Others have suggested that IFN-α therapy may in fact lead to a decline in HCV-specific T cell responses, but not in T cells targeting non-HCV antigens, and that these responses remain undetectable in those with an SVR, suggesting that the decline in HCV-specific response mirrors the fall in HCV viral load.^44^

In this study of genotype-3a infection we show that HCV-specific T cell responses that are detectable before therapy decline significantly during therapy in individuals with a subsequent SVR, but recover once IFN is stopped. In patients who do not have an SVR the decline in HCV-specific responses is not apparent. However, the same pattern is also observed in non-HCV T cell responses suggesting a generic host response to IFN treatment. Moreover, when these observations are extended to include total lymphocyte counts it becomes apparent that in genotype-3a infection lymphopenia is specifically associated with an SVR to combination therapy whereas treatment NRs are resistant to treatment induced lymphopenia. The mechanism of IFN induced lymphopenia is not known but may represent T cell redistribution. The fact that total lymphocytes and non-HCV-specific T cells are reduced specifically in those with a subsequent SVR, but recover after treatment stops in spite of undetectable viraemia, suggest that the decline in HCV-specific T cell responses has little to do with changes in HCV viral load. More likely, genotype-3a infected hosts who are treatment non-responsive show evidence of a general host resistance to the effects of IFN therapy. Recent data showing that genetic polymorphisms linked to the IL28B gene determine the outcome of treatment with IFN therapy strongly supports the concept that host genetics play a key role in the clinical outcome of infection. It may be that IFN stimulated genes induce peripheral lymphopenia and also successfully eradicate virus in the host. Certainly, there are data supporting the idea that IFN stimulated genes are stimulated in PBMC in IFN treated people.^45^ Alternatively, a preactivated IFN system induced by viral proteins, which is known to be associated with a failure to respond to exogenous IFN, may somehow render the host resistant to exogenous IFN induced lymphopenia.

In conclusion, our data demonstrate that genotype-3a HCV exhibits a distinct T cell specificity. In chronic infection, CD4 T cells that target the structural proteins and CD8 T cells that target the NS proteins are readily detectable, while CD4 T cells that target the NS proteins are associated with spontaneous viral control. Viral sequence analysis showed evidence of viral escape from CD8 T cells but not CD4 T cells. Paradoxically, a successful treatment outcome in genotype-3a infection was associated with a decline in HCV and non-HCV-specific T cells and with a decline in the total lymphocyte count.
